# Assessment of trunk and shoulder muscle asymmetries during two-armed kettlebell swings: implications for training optimization and injury prevention

**DOI:** 10.3389/fspor.2024.1497826

**Published:** 2024-11-05

**Authors:** Khaled Abuwarda, Abdel-Rahman Akl

**Affiliations:** ^1^ Department of Physical Education and Kinesiology, College of Education, Qassim University, Qassim, Saudi Arabia; ^2^ Faculty of Physical Education-Abo Qir, Alexandria University, Alexandria, Egypt

**Keywords:** physical activity, strength training, wearable sensors, EMG, sports exercise

## Abstract

**Introduction:**

Greater side-to-side asymmetry can indicate impaired skill, reduced power production, and an increased risk of injury. Bilateral differences highlight the presence of asymmetries that should be assessed to understand their impact on both injury risk and performance enhancement.

**Objective:**

This study aimed to assessment muscle activation and bilateral asymmetry in major trunk and shoulder muscles during a two-armed kettlebell swing exercise.

**Methods:**

Twenty-seven participants (age: 24.2 ± 2.6 years; body mass: 82.9 ± 7.7 kg; height: 176.9 ± 7.0 cm) were included in the study. Electromyographic (EMG) data were collected bilaterally from twelve muscles (six muscles per side: anterior deltoid [AD], posterior deltoid [PD], erector spinae longissimus [ESL], erector spinae iliocostalis [ESI], external oblique [EO], and rectus abdominis [RA]).

**Results:**

Results indicated that asymmetry indices for the AD, ESL, ESI, and RA muscles during the upward propulsion phase fell within the determined threshold of 15%. However, the asymmetry indices for the PD and EO muscles exceeded this threshold by 3.36% and 2.62%, respectively. The findings suggest that trunk muscle asymmetries during the kettlebell swing are generally less pronounced than those of the shoulder muscles, particularly during the float phase.

**Conclusion:**

These results provide valuable insights into bilateral muscle asymmetry during a two-armed kettlebell swing, which can inform the development of targeted training programs. The methods and findings of this study may further contribute to understanding the effects of muscle balance, symmetry, and injury mechanisms in dynamic movements.

## 1 Introduction

Over the last decade, resistance training has gained popularity as a form of exercise due to its ability to enhance athletic performance in various ways (1). Among these methods, kettlebell training has garnered increasing interest and popularity among both professional and amateur athletes since its introduction in 2009 (2).

Kettlebell training is a versatile resistance-training technique that serves multiple purposes, including improving muscular strength (3), endurance (1), explosive power (4), weight management and flexibility (2), general fitness (5), and injury rehabilitation (6). Kettlebells have been incorporated into strength and conditioning programs across various sports, such as handball (7), shot put (8), sprinting (9), and soccer (10). Previous research has demonstrated that kettlebells can enhance 1-RM in back squats (3), increase power output in vertical jumps and power (11), and improve aerobic capacity in female soccer players (6, 12). Additionally, kettlebells have been considered for military task performance (4, 13) and as part of the Royal Air Force's aircrew training program (14). They are also used in clinical settings for osteoporosis management, fall and fracture prevention (15), and fitness enhancement among healthcare workers (16), as well as in initiatives aimed at improving health-related physical fitness (4, 17).

Traditionally, kettlebells are made of cast iron and increase in size with weight. Currently, they are available in various materials and weights, ranging from 2 kg to 92 kg (4). The six fundamental hardstyle techniques include the Turkish get-up, clean, swing, squat, press, and snatch and the two-handed hardstyle swing was identified as the most frequently used kettlebell exercise (4). This movement involves propelling the kettlebell forward and upward with a swinging motion driven by hip extension, transferring force to the trunk and arms (6).

Several studies have examined surface electromyography-based muscle activation during the kettlebell swing exercise (5, 6, 18). Research has focused on muscles most affected by the exercise, including the anterior deltoid, erector spinae, rectus abdominis, and external oblique (18). Van Gelder, Hoogenboom (19) demonstrated that both one-handed and two-handed kettlebell swings effectively recruit muscles for strengthening. Andersen, Fimland (20) specifically investigated trunk muscle activation during one-handed vs. two-handed Russian kettlebell swings. Assessing muscle activity across various kettlebell swing variations is crucial for understanding the exercise's potential applications in injury prevention, performance enhancement, and rehabilitation (5).

Previous research has shown that unilateral and bilateral resistance training exercises have distinct effects on trunk muscle activation (21, 22). The kettlebell swing can be performed either unilaterally or bilaterally (6). Bilateral asymmetry, defined as differences in function or performance between the dominant and non-dominant limb or side, is an important factor in assessing injury risk and performance enhancement (23). The asymmetry index (AI) is used to measure imbalances be-tween the dominant and non-dominant limbs or sides during performance (24). Prolonged engagement in the same sport can lead to the development of bilateral asymmetry (25, 26), which is increasingly recognized as a concern due to its impact on the likelihood of sports injuries (27).

Current literature suggests that multiple evaluations of asymmetry in a given activity may re-veal varying degrees of imbalance, often without consistently favoring the same side or direction (28). Madruga-Parera, Bishop (29) examined whether asymmetry remained consistent across three unilateral jump-based assessments among team sport participants. For advanced lifters, who are accustomed to lifting loads closer to their 1RM, it is beneficial to analyze whether weight distribution asymmetries persist in individuals with high technical proficiency (30).

Greater side-to-side asymmetry may indicate impaired skill and power production, along with a heightened risk of injury (31). A symmetry score of zero indicates perfect weight distribution, while scores above zero suggest varying degrees of asymmetry, with higher AI scores representing greater imbalance (30). To minimize injury risk and achieve optimal performance, it is crucial to reduce performance imbalances (32, 33). Bilateral asymmetry in functional performance above a specific threshold has been associated with increased sports-related injury risk (34). Thus, measuring bilateral asymmetry in physical, biomechanical, and electromyographical variables can be critical in athlete evaluation (35), and provide a valuable monitoring tool (36). Consistency across these measures, in addition to assessing bilateral asymmetry, could offer insights for targeted exercise interventions tailored to each individual's dominant and non-dominant sides (37, 38).

Therefore, studying the factors influencing muscle activation and bilateral asymmetry during the two-armed kettlebell swing can provide valuable insights into its effectiveness, potentially enhancing our understanding of performance improvement and injury mechanisms. The current study aimed to identify differences in muscle activation and bilateral asymmetry of major trunk and shoulder muscles on both sides during the two-armed kettlebell swing exercise. We hypothesized that bilateral asymmetry would score low, indicating near-perfect symmetry in weight distribution according to the established threshold.

## 2 Materials and methods

### 2.1 Subjects and study design

A priori power analysis was calculated using G-Power software version 3.1.9.7 (Universität Kiel, Germany). Assuming 6 repetitions per subject, a power of 0.80, an alpha level of 0.05, and a medium effect size of 0.25, indicated that an average of 19 participants would be needed for statistical tests. Based on this estimate, 27 participants were included in the study (age: 24.2 ± 2.6 years; body mass: 82.9 ± 7.7 kg; height: 176.9 ± 7.0 cm). The inclusion criteria required participants to be over 18 years old and have at least five years of experience in kettlebell training. Participants were excluded if they had significant gaps in their training history (longer than six months) or if they were currently injured or recovering from injuries that could affect their performance. All participants provided written informed consent, and the study was approved by the institution's ethics committee.

### 2.2 Experiment protocol

**Figure 1:**
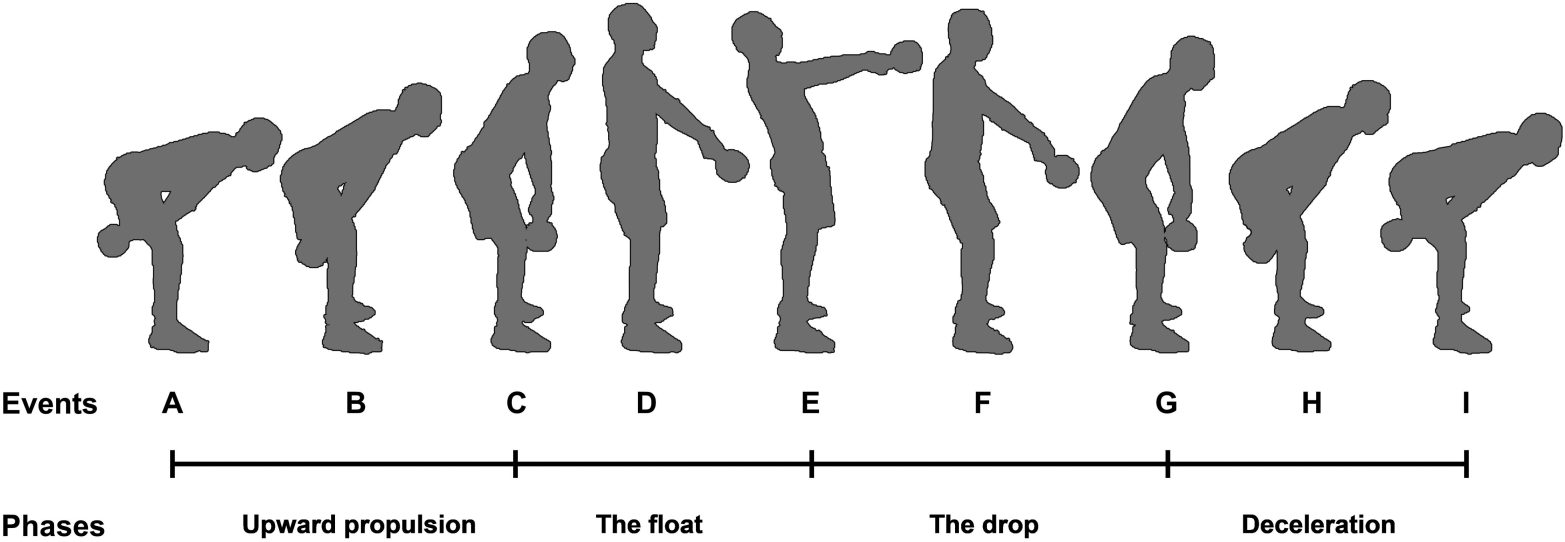
A single cycle of two-armed kettlebell swing phases: **(A**–**C)** phase 1 (upward propulsion of the kettlebell), **(C**–**E)** phase 2 (the float: passive shoulder flexion with hips and knees in terminal extension), **(E)** Mid-swing (top of the swing), (E-G) phase 3 (the drop: passive extension of the shoulders with hips and knees in terminal extension), **(G**–**I)** phase 4 [deceleration of the kettlebell to the end of the cycle (bottom of the swing)].

Participants performed a kettlebell-specific warm-up that included five minutes of submaximal kettlebell swings. Following the warm-up, they completed one set of two-armed Russian kettlebell swings, with each set consisting of five repetitions using a 16-kg kettlebell. The sequence of the exercises remained consistent between the warm-up and the experimental tests. The participants’ self-selected foot stance was recorded during the initial exercise and maintained throughout all tests and the participants were asked which limb they would prefer to use for the exercises in order to identify which limb was dominant (39). Using a Simi Motion Capture System (Simi Reality Motion Analysis V. 9.0.6) that was synchronized with the EMG at 100 frames per second, video analysis was used to determine the phases; swing phases and timing were measured upon the participant's signal, with the swing divided into four distinct phases: upward propulsion, float, drop, and deceleration (Figure 1).

**Figure 2:**
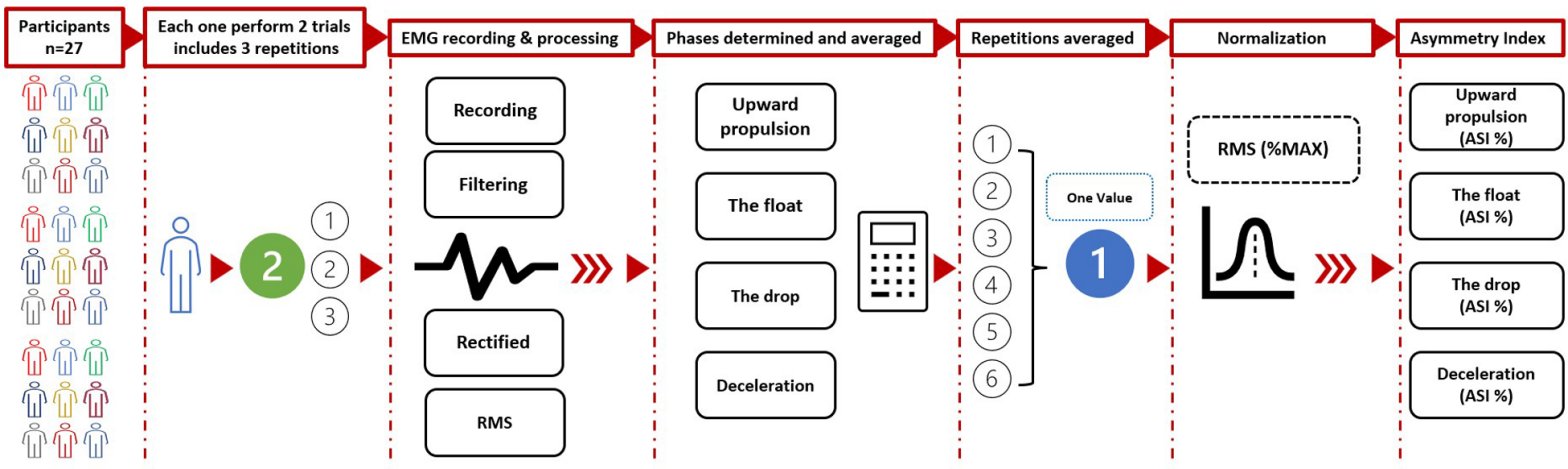
Flow diagram of the test and processing procedures.

Participants initiated the swing by flexing their hips during the downward movement and ex-tending them rapidly and forcefully to lift the kettlebell to chest height in a “Russian swing” motion (4). Each participant completed two trials of the two-armed kettlebell swings, performing at least five repetitions per trial with the 16 kg kettlebell (40). To avoid fatigue, a one-minute rest period was provided between trials. The first and last repetitions were excluded from analysis. Results from the successful trials (two trials with three repetitions each) were averaged to generate a single variable for each participant, which was then used in the asymmetry index calculation and statistical analyses (Figure 2) (41).

### 2.3 sEMG activity recording and analysis

In accordance with SENIAM guidelines, the participants’ skin was shaved, abraded, and cleaned with alcohol prior to electrode application (42). Gel-coated, self-adhesive electrodes (bipolar, 10 mm diameter silver chloride surface electrodes; SKINTACT FS-RG1/10, Leonhard Lang GmbH, Innsbruck, Austria) were used, with a 2 cm center-to-center spacing. Electrodes were placed according to SENIAM guidelines (www.seniam.org) on selected trunk and upper limb muscles: rectus abdominis (RA), external oblique (EO), erector spinae longissimus (ESL), erector spinae iliocostalis (ESI), anterior deltoid (AD), and posterior deltoid (PD) on both dominant and non-dominant sides. A surface EMG device (Myon m320RX; Myon, Switzerland) recorded the raw EMG signals, which were sampled using a 16-bit A/D converter at 1,000 Hz. EMG data were processed using Visual 3D software (C-Motion, Germantown, MD, USA). To reduce movement artifacts, a high-pass Butterworth filter with a cut-off frequency of 25 Hz and a low-pass filter at 15 Hz were applied. The signals were preprocessed using a full-wave rectiﬁer and a linear envelope obtained using the root mean square (RMS) approach with a window size of 100 ms (43). EMG signal amplitudes were normalized to the maximum signal observed across the two trials, including all three repetitions for each participant.

### 2.4 Asymmetry index

The asymmetry Index was calculated following the methodology of Sheikhi, Letafatkar (44), measures the degree of bilateral distribution symmetry across the upper limbs and trunk muscles (Equation 1).

(1) $$\mathrm{Asymmetry}\;\mathrm{Index}\;(\text{\%})=\frac{2(\mathrm{dominant}\;-\;\mathrm{non}\;\mathrm{dominant}\;)\;}{\left(\mathrm{dominant}\;+\;\mathrm{non}\;\mathrm{dominant}\;\right)}*100$$

**Figure 3:**
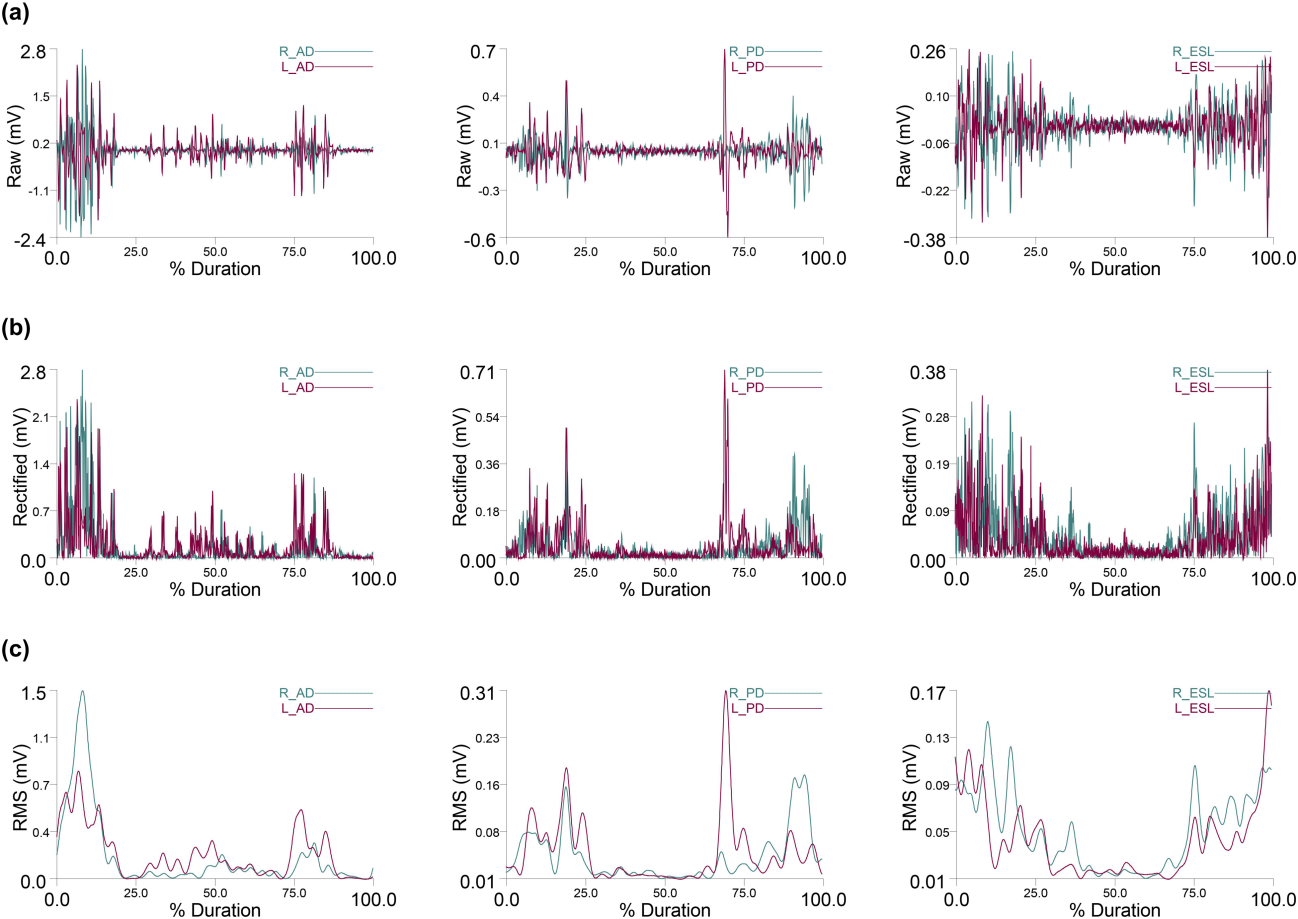
Anterior deltoid (AD), posterior deltoid (PD), and erector spinae longissimus (ESL) muscle activity. **(a)** Raw EMG data, **(b)** Rectified EMG data, and **(c)** data from a representative subject.

**Figure 4:**
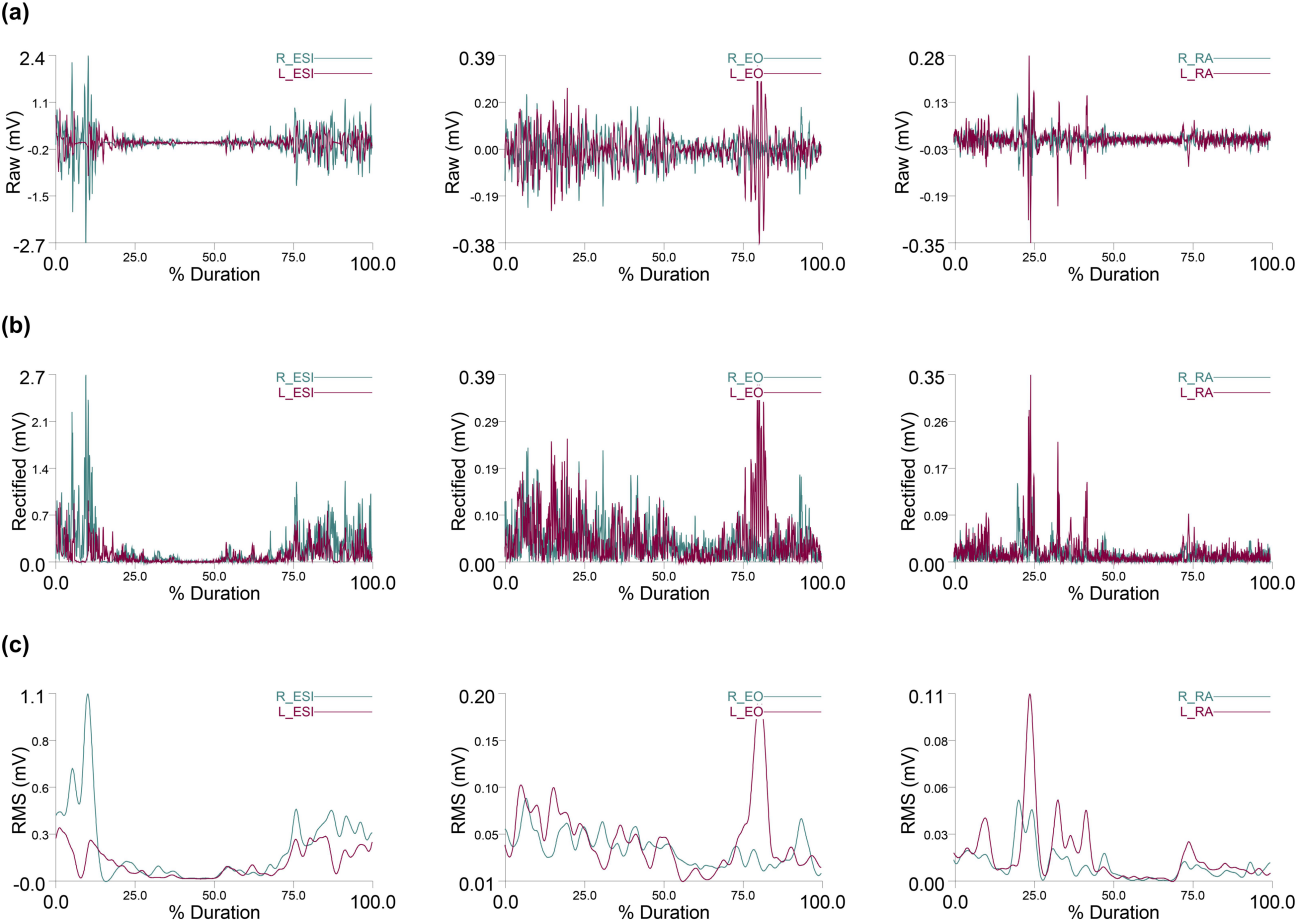
Erector spinae iliocostalis (ESI), external oblique (EO), and rectus abdominis (RA) muscle activity. **(a)** Raw EMG data, **(b)** Rectified EMG data, and **(c)** data from a representative subject.

An index value of zero represents perfect symmetry, while values greater than zero indicate varying degrees of asymmetry, with larger values signifying more pronounced asymmetry (30). The Asymmetry Index was calculated for each muscle on both sides based on muscle activity during the two-armed kettlebell swing. Figures 3, 4 display the raw, rectified, and RMS values of muscle activation from a representative subject.

### 2.5 Statistical analysis

Descriptive statistics were reported as means and 95% confidence intervals (mean ± CI). Data distribution was assessed using Shapiro-Wilk tests, confirming the suitability for parametric analysis. Within-group differences between phases were evaluated using repeated measures one-way analysis of variance (RM ANOVA), with Sidak *post hoc* tests comparing the means of each variable during the four phases (upward propulsion, float, drop, and deceleration). Effect size was assessed using partial eta squared (*η*^2^_p_), where *η*^2^_p_ ≥ 0.01 is a small effect, *η*^2^_p_ ≥ 0.06 is a medium effect, and *η*^2^_p_ ≥ 0.14 is a large effect (45). All statistical analyses were conducted using IBM SPSS Statistics v27 (IBM® Corp., NY, USA).

## 3 Results

**Figure 5:**
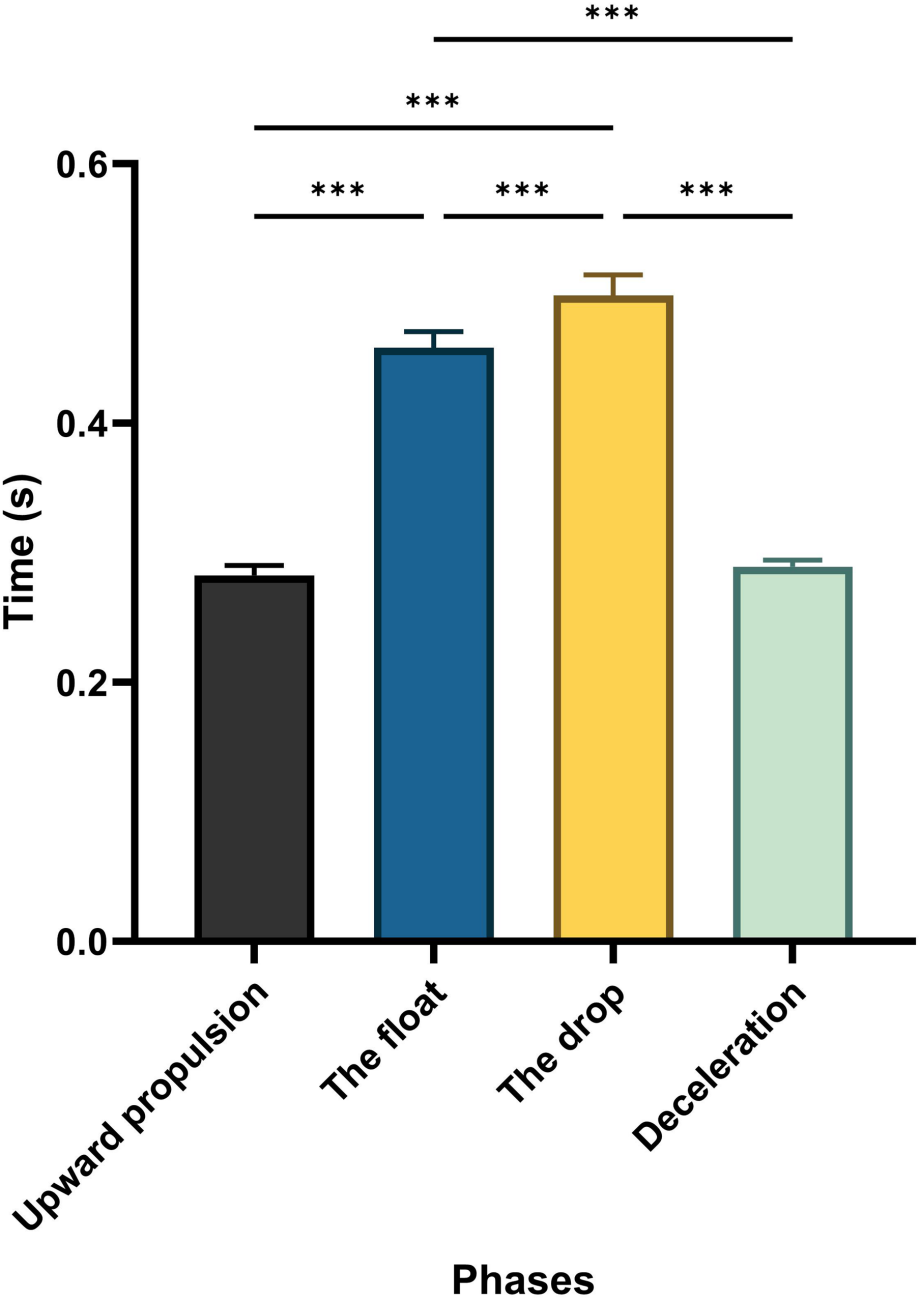
Average values and CI for the duration for the two-armed kettlebell phases. Significant differences for the *post hoc* tests between phases: Partial eta squared (*η*^2^_p_ = 0.97) and asterisk signs represent significant differences between speeds: (***) indicates *p* < 0.001.

Figure 5 displays the average values and confidence intervals for the duration of the two-armed kettlebell swing phases. The RM-ANOVA showed significant main effects across the exercise phases (*η*^2^_p_ = 0.97). *post hoc* comparisons indicated significant differences among the phases (*p* < 0.001), with the longest duration observed during the drop phase, followed by a decrease in the deceleration phase.

**Figure 6:**
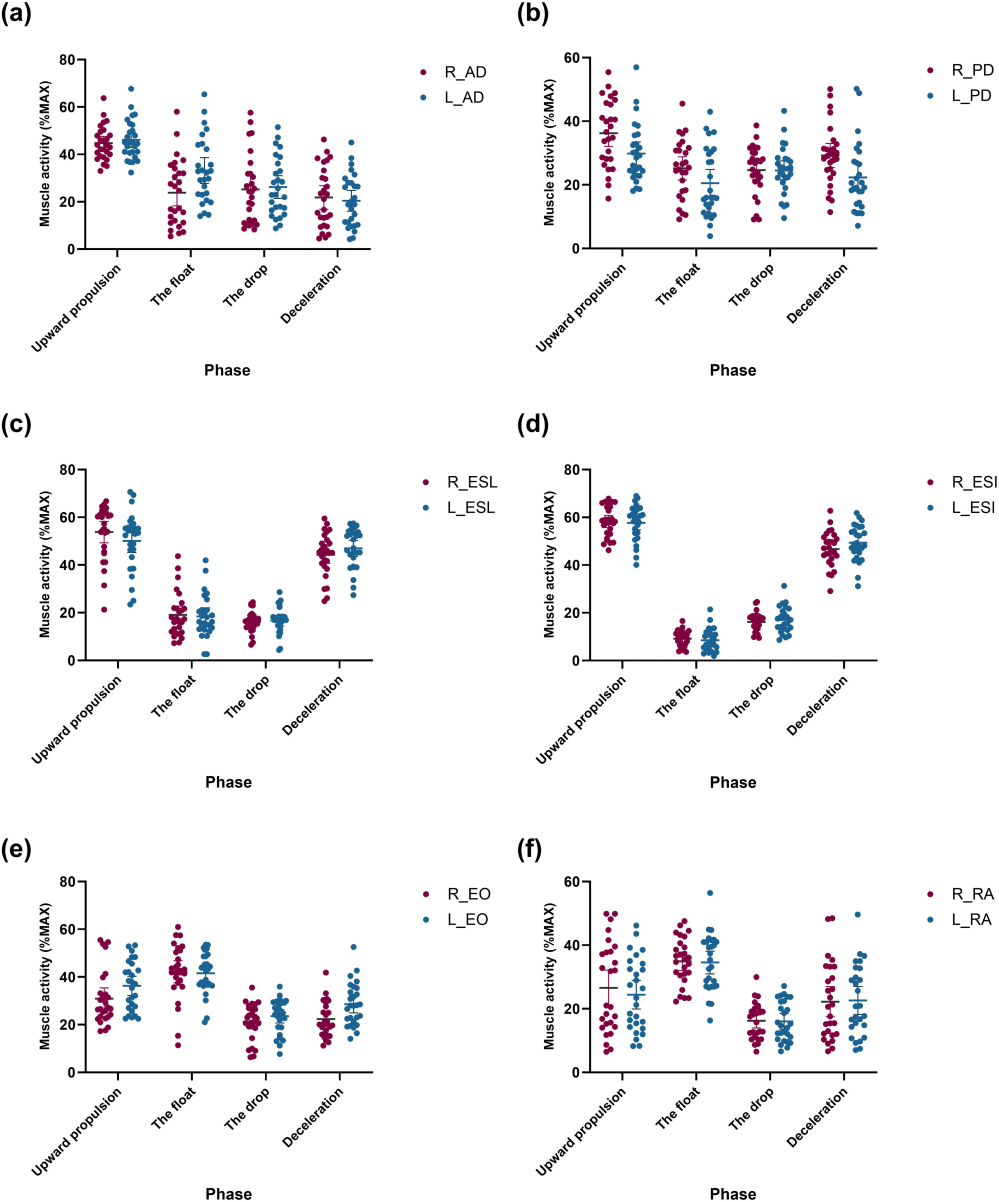
Average values and CI for the normalized RMS muscles activation (%MAX) per phase of the two-armed kettlebell exercise: **(a)** right and left AD muscles, **(b)** right and left PD muscles, **(c)** right and left ESL, **(d)** right and left ESI muscles, **(e)** right and left EO muscles, and **(f)** right and left RA muscles.

Figure 6 presents the average values and confidence intervals for the normalized RMS (%max) of the right and left limbs during the phases of the two-armed kettlebell swing. Muscle activities of the right and left anterior deltoid (AD), posterior deltoid (PD), erector spinae longissimus (ESL), erector spinae iliocostalis (ESI), external oblique (EO), and rectus abdominis (RA) were recorded during the upward propulsion, float, drop, and deceleration phases, respectively (Figure 6). The peak activities of the right and left AD, PD, ESL, and ESI muscles occurred during the upward propulsion phase, while the peak activities of the right and left EO and RA muscles occurred during the float phase.

**Figure 7:**
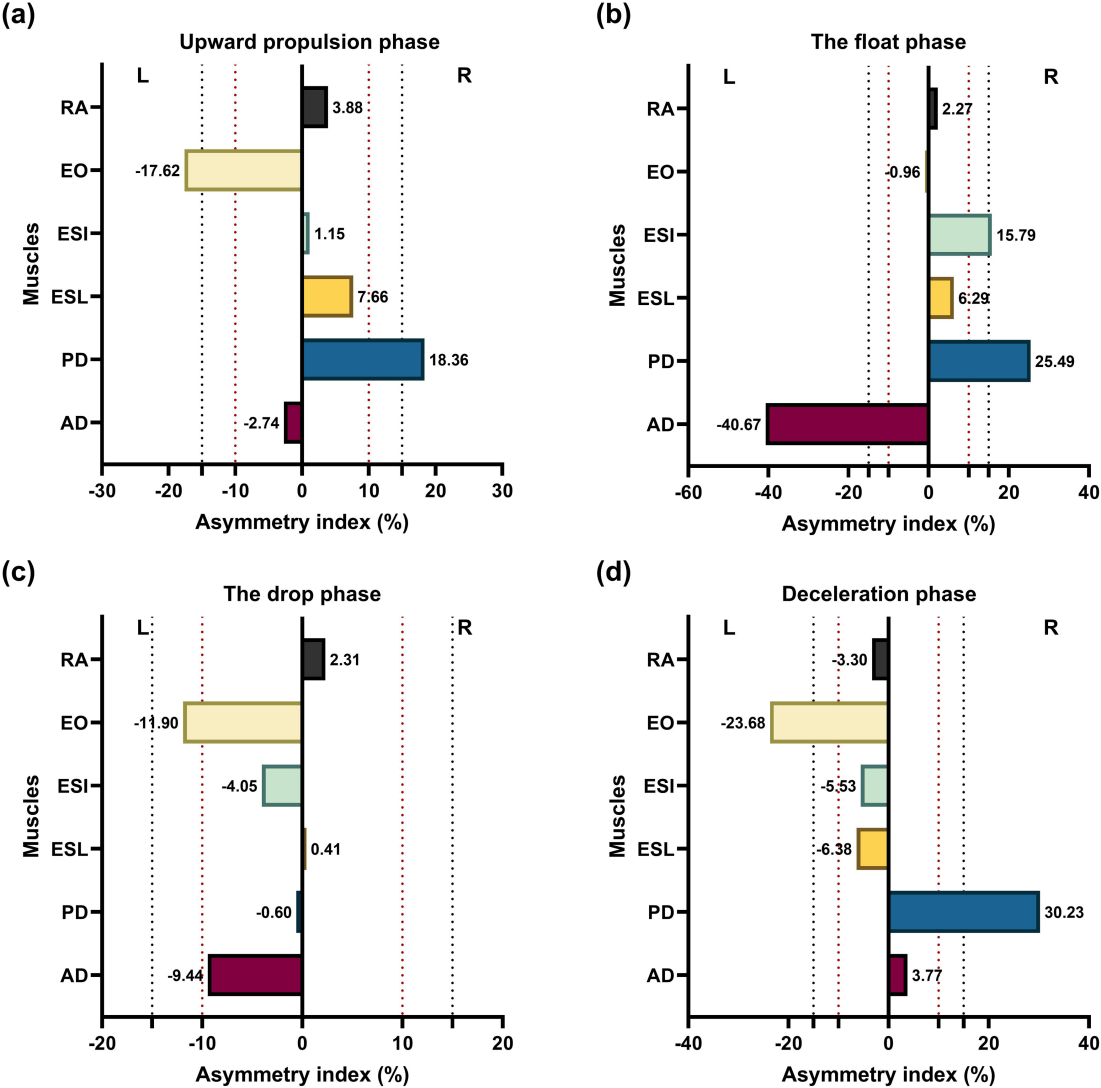
Muscles asymmetry data (%) for two-armed kettlebell exercise during the four phases: **(a)** upward propulsion, **(b)** the float, **(c)** the drop, and **(d)** deceleration. The asymmetry favoring the right side was indicated by bars oriented towards the right, while the asymmetry favoring the left side was indicated by bars oriented towards the left. Dot lines indicate; red 10% and black 15% of asymmetry threshold for right and left (L) side.

Figure 7 provides the asymmetry indices for individual muscles during each phase. During upward propulsion, asymmetry indices were AD (−2.74%), PD (18.36%), ESL (7.66%), ESI (1.15%), EO (−17.62%), and RA (3.88%) (Figure 7a). During the float phase, asymmetry indices were AD (−40.67%), PD (25.49%), ESL (6.29%), ESI (15.79%), EO (−0.96%), and RA (2.27%) (Figure 7b). During the drop phase, asymmetry indices were AD (−9.44%), PD (−0.60%), ESL (0.41%), ESI (−4.05%), EO (−11.90%), and RA (2.31%) (Figure 7c). During deceleration, asymmetry indices were AD (3.77%), PD (30.23%), ESL (−6.38%), ESI (−5.53%), EO (−23.68%), and RA (−3.30%) (Figure 7d).

**Figure 8:**
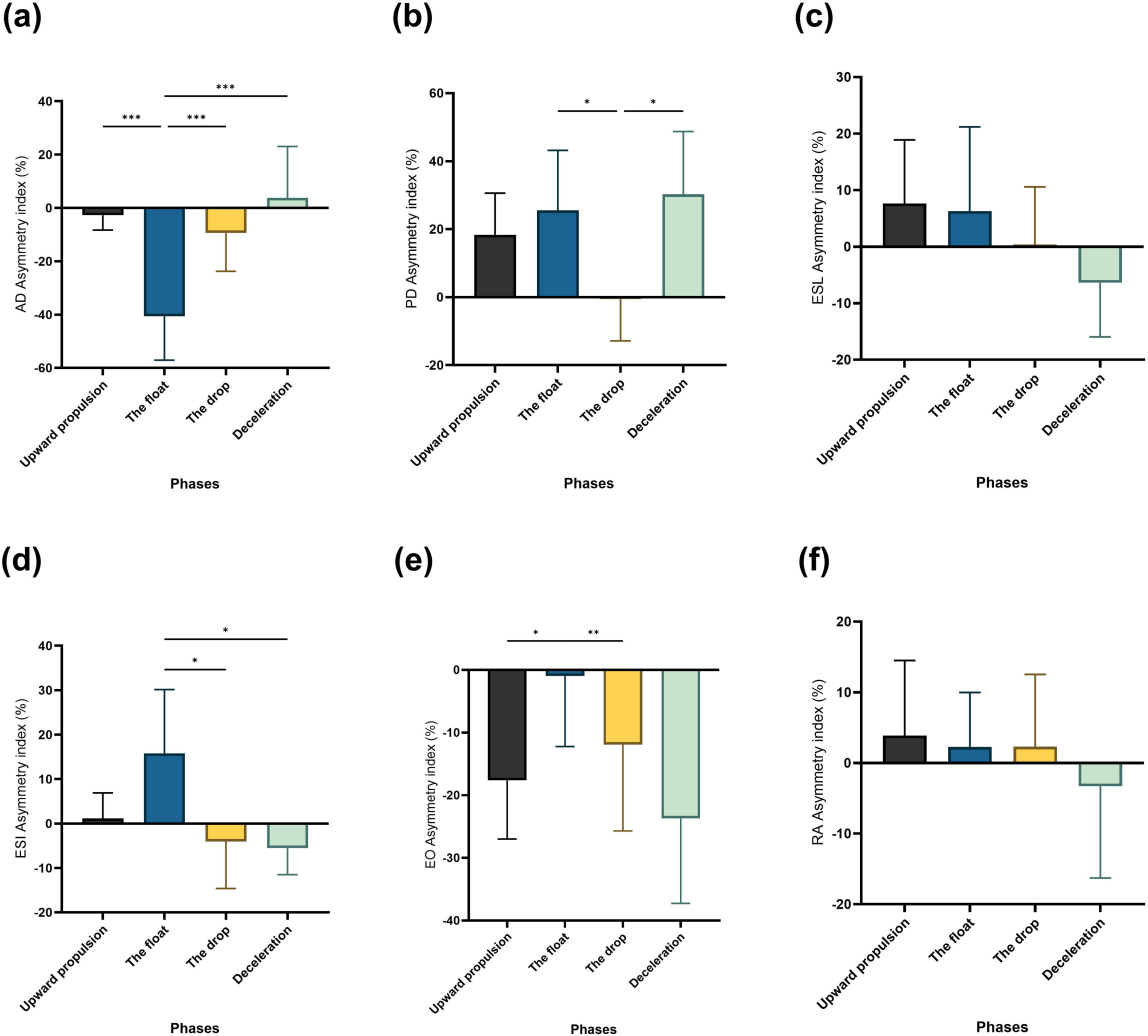
Pairwise comparisons associated with the significant main effects from the RM-ANOVA with mean and confidence intervals for the asymmetry index (%) per phase: **(a)** AD bilateral asymmetry, **(b)** PD bilateral asymmetry, **(c)** ESL bilateral asymmetry, **(d)** ESI bilateral asymmetry, **(e)** EO bilateral, and **(f)** RA bilateral asymmetry. Asterisk signs represent significant differences between speeds: (***) indicates *p* < 0.001, (**) indicates *p* < 0.01, (*) indicates *p* < 0.05.

The RM-ANOVA revealed significant main effects for bilateral asymmetries in AD (F = 12.71, *η*^2^_p_ = 0.33) (Figure 8a), PD (F = 4.52, *η*^2^_p_ = 0.15) (Figure 8b, ESL (F = 1.70, *η*^2^_p_ = 0.06) (Figure 8c), ESI (F = 5.45, *η*^2^_p_ = 0.17) (Figure 8d), EO (F = 4.30, *η*^2^_p_ = 0.14) (Figure 8e), and RA (F = 0.79, *η*^2^_p_ = 0.03) (Figure 8f). Significant differences in asymmetries were found between upward propulsion and float phases for AD (*p* < 0.001) and EO (*p* < 0.05), but not for PD, ESL, ESI, or RA. Additional significant differences were observed between the float and drop phases for AD (*p* < 0.001), PD (*p* < 0.05), ESI (*p* < 0.05), and EO (*p* < 0.01). For the drop and deceleration phases, significant variations were found only for PD (*p* < 0.05). Lastly, significant differences were identified between the float and deceleration phases for AD (*p* < 0.001) and ESI (*p* < 0.05).

## 4 Discussion

The aim of this study was to identify differences in muscle activation and examine bilateral asymmetry in the major trunk and shoulder muscles during the two-armed kettlebell swing exercise. To our knowledge, this is the first study to investigate muscle activity asymmetry during the two-armed kettlebell swing using electromyographic analysis. This approach provides insights into the effective-ness of this technique, contributing to the understanding of performance enhancement and potential injury mechanisms.

Based on the bilateral performance of the two-armed kettlebell swing, we hypothesized that muscle activity would be symmetrical between sides. However, the results revealed that the AD muscle on the left side exhibited greater activation compared to the right, particularly during the float phase. The discrepancy between the sides diminished during the drop and deceleration phases. This finding suggests that the AD muscle plays a key role in accelerating the kettlebell specifically during the initial upward movement, which is in agreement with Salem, Hassan (18). During the two-armed kettlebell swing phases, the PD muscles on both sides exhibited a high level of asymmetry (>15%), resulting in notable differences between the dominant and non-dominant sides during the upward propulsion, float, and deceleration phases. In contrast, the PD muscle's primary role is to engage in braking movement during the drop phase, leading to an asymmetry level of less than 15% in this phase.

Simultaneous activity of the trunk muscles (ESL, ESI, EO, and RA) on both sides, especially during the upward propulsion, float, and drop phases, underscores the importance of stability in the two-armed kettlebell swing. In general, and particularly in sports, greater joint coordination instability increases the risk of injury, while smaller side-to-side asymmetry helps reduce this risk and enhances training effectiveness (36, 46). Several studies have shown that higher functional asymmetries are associated with an increased risk of injury and are considered a known risk factor (30, 47). Thus, reducing asymmetry in these muscles can enhance performance and lower the risk of injury (48, 49).

To evaluate bilateral muscle activity differences, we employed the asymmetry index (AI) method to calculate the asymmetry between the right and left sides for each muscle. Our findings, along with previous studies, suggest a 15% threshold of inter-limb or side asymmetry as normal physiological variability, serving as a reference value for injury risk (dotted lines in Figure 7) (50–52). This study is the first to quantify and compare muscle activity asymmetry during and between phases of the two-armed kettlebell swing exercise. Regarding muscle asymmetry, our findings indicate that the asymmetry indexes for AD, ESL, ESI, and RA muscles during the upward propulsion phase were within the acceptable range of 15%. These results suggest that the observed asymmetries are within normal limits during performance. However, the PD and EO muscles exhibited asymmetries exceeding the targeted threshold, possibly due to unilateral activation or compensatory responses aimed at enhancing stability. The ESL, ESI, and RA muscles consistently remained within the AI threshold across all four phases, likely due to the RA's role in core stability, facilitating force transfer from the hip extensors to the arms and kettlebell. Additionally, trunk muscles like the ESL are crucial for extending the trunk during the upward phase and providing stability to prevent spinal twisting under load (20). Extensor core muscles are activated in response to flexion moments, further supporting their stabilizing role (53).

Our results support the hypothesis that bilateral asymmetry would be minimal, indicating near-optimal weight distribution symmetry based on the established threshold. However, the PD muscle demonstrated high asymmetry during the upward propulsion, float, and deceleration phases, while the AD muscle showed significant asymmetry during the float phase. Additionally, the EO muscle exhibited slightly greater asymmetry than the threshold during the upward propulsion and deceleration phases. This highlights the critical roles of these muscles in kettlebell training. The AD muscle generally shows greater activation than the PD during kettlebell exercises, with both muscles being essential for controlling the swing, especially during the float phase (18). These asymmetry index results are valuable for physiotherapists and strength and conditioning coaches in identifying athletes at risk of injury, enabling the development of targeted neuromuscular prevention programs. Corrective strategies focused on reducing asymmetry may help lower the risk of injury (50).

Analyzing inter-phase asymmetry revealed significant differences in the AD muscle between phases, with notable asymmetry during the float phase, likely due to the increased load of resisting the kettlebell's weight and gravity. Additionally, differences in bilateral asymmetry were observed between the float and drop, and float and deceleration phases for the PD, ESI, and EO muscles. Our findings suggest that trunk muscle asymmetries during the two-armed kettlebell swing are generally lower than those observed in shoulder muscles (AD and PD), particularly during the float phase, which plays a key role in generating muscle activity and force during the ballistic lift of the kettlebell.

## 5 Practical applications

The study's findings have practical implications for coaches and practitioners aiming to optimize kettlebell training. By identifying phases with higher asymmetry, targeted corrective exercises can be incorporated into training routines to enhance symmetry and reduce injury risk. Moreover, the use of sEMG to monitor muscle activation and asymmetry provides a valuable tool for assessing training progress and individualizing exercise prescriptions.

## 6 Limitations and future work

Despite the robust findings, this study has some limitations. The relatively small sample size may limit the generalizability of the results. Future studies should include a larger cohort to confirm these findings. Additionally, while this study focused on trunk and upper limb muscles, lower limb muscle activity, which also plays a crucial role in kettlebell swings, was not assessed. Incorporating lower limb muscles into the analysis could provide a more comprehensive understanding of the exercise's demands. Lastly, exploring the impact of different kettlebell weights and swing variations could further elucidate the relationship between load, phase duration, and muscle asymmetry.

## 7 Conclusions

This study concluded that asymmetry indices for the anterior deltoid (AD), erector spinae longissimus (ESL), erector spinae iliocostalis (ESI), and rectus abdominis (RA) muscles during the upward propulsion phase were within the determined threshold of 15%. However, the asymmetries of the posterior deltoid (PD) and external oblique (EO) muscles exceeded this threshold by 3.36%–2.62%, respectively. The results suggest that trunk muscle asymmetries during the two-armed kettlebell swing are generally smaller than those of the shoulder muscles (AD and PD), particularly during the float phase. These findings highlight the importance of monitoring bilateral muscle activity and addressing asymmetries when performing the two-armed kettlebell swing exercise. Trainers and professionals should consider these asymmetries to reduce imbalances between both sides, potentially enhancing performance and reducing injury risk. This study provides valuable insights into muscle bilateral asymmetry during kettlebell swings, which can inform the development of targeted training programs. The techniques and findings presented may further our understanding of muscle balance, symmetry, and injury mechanisms in dynamic exercises. Ultimately, the application of these insights will need to be tailored to each individual, requiring personalized assessment by coaches and medical professionals to effectively address muscle asymmetries and optimize training outcomes.

## Data Availability

The raw data supporting the conclusions of this article will be made available by the authors, without undue reservation.
