# Corrigendum: Assessment of trunk and shoulder muscle asymmetries during two-armed kettlebell swings: implications for training optimization and injury prevention

**DOI:** 10.3389/fspor.2024.1535151

**Published:** 2024-12-09

**Authors:** Khaled Abuwarda, Abdel-Rahman Akl

**Affiliations:** ^1^ Department of Physical Education and Kinesiology, College of Education, Qassim University, Qassim, Saudi Arabia; ^2^ Faculty of Physical Education-Abo Qir, Alexandria University, Alexandria, Egypt

**Keywords:** physical activity, strength training, wearable sensors, EMG, sports exercise

A Corrigendum on Assessment of trunk and shoulder muscle asymmetries during two-armed kettlebell swings: implications for training optimization and injury prevention By Abuwarda K and Akl A-R (2024). Front Sports Act Living. 6.1497826 doi: 10.3389/fspor.2024.1497826

**Incorrect Correspondence**

In the published article, there was an error in Correspondence Abdel-Rahman Akl. Instead of “Abdel-Rahman Akl: abdelrahman.akl@alexu.edu.eg”, it should be “Abdel-Rahman Akl: abdelrahman.akl@alexu.edu.eg and Khaled Abuwarda: k.libda@qu.edu.sa”.

The authors apologize for this error and state that this does not change the scientific conclusions of the article in any way. The original article has been updated.

